# Accelerated evolution increased craniofacial divergence between humans and great apes

**DOI:** 10.1098/rspb.2025.1507

**Published:** 2025-10-22

**Authors:** Aida Gómez-Robles, Amy Drennan, Maricci Basa, Alfie Gleeson

**Affiliations:** ^1^ Department of Anthropology, University College London, London, UK; ^2^ Department of Cell and Developmental Biology, University College London, London, UK; ^3^ Department of Genetics, Evolution and Environment, University College London, London, UK; ^4^ Bat Conservation Trust, London, UK

**Keywords:** hominoids, hominids, great apes, hylobatids, gibbons, phylogeny, geometric morphometrics, cranium

## Abstract

The level of craniofacial diversity of hominids (the group that includes great apes and humans) is much higher than that of their sister group, the hylobatids (also known as gibbons or lesser apes), despite the similar timeline of diversification and a similar level of genetic differentiation between the two clades. To shed light on the evolutionary dynamics shaping these varying levels of craniofacial diversity, we used three-dimensional high-density geometric morphometric approaches and phylogenetic comparative methods. We show that neurocranial diversity exceeds that expected from neutral evolution in the great apes with respect to the gibbons, whereas facial diversity does not. These results indicate that neurocranial diversity across the great apes has been shaped by genus-specific neurocognitive, social or ecological selective pressures that are not observed in the gibbons, whose neurocranial diversity is constrained by stabilizing selection and gene flow. However, facial diversity results from similar evolutionary dynamics across both clades. Within this general pattern of differences and similarities between the great and lesser apes, humans emerge as the only species that consistently shows the highest evolutionary rate across almost all craniofacial regions in both males and females, thus pointing to strong human-specific selective pressures in neurocranial and facial evolution.

## 1. Introduction

Human craniofacial anatomy differs substantially from that of the great apes. While the great apes have big and forwardly projecting faces and relatively small brains, humans are characterized by an orthognathic face and a globular neurocranium [1]. Some researchers consider that human craniofacial anatomy has evolved in response to the primary selective pressure on increasing overall brain size and the size of particular brain regions [2–4], but others consider that human neurocranial shape has evolved as a result of integration patterns linking a flatter face with a more globular neurocranium [5,6]. Interestingly, human craniofacial anatomy appears more similar to that of the lesser apes (also known as hylobatids or gibbons), who also have a relatively flat face and a more globular braincase [7–9], than to the great apes’, despite the greater evolutionary distance separating humans and gibbons. It has been suggested that patterns of morphological integration between the face and the neurocranium have made gibbons and humans evolve in similar ways despite their large evolutionary distance [9].

Within hominoids (the clade including great and lesser apes), hominids (great apes and humans) show a large degree of anatomical diversity, spanning from small-faced and large-brained humans to large-faced and relatively small-brained gorillas. Hylobatids, however, show a low level of craniofacial diversity across species, with some species virtually indistinguishable based on craniofacial morphology, even when they show differences in their pelage, vocalizations, external features and karyotypes, among others [8,10,11]. Anatomical, genetic and biogeographical comparisons indicate that gibbons experienced rapid evolutionary radiation, hence their overall anatomical similarity [9,11,12]. This radiation is inferred to have happened approximately 7 million years ago (Ma), which coincides with the inferred divergence time between the chimpanzee and human lineages [12]. Also, genetic differentiation across all hylobatid genera is similar to the level of genetic differentiation between chimpanzees and humans [13]. However, chimpanzees and humans differ in craniofacial morphology substantially more than any two species of gibbons, so it is not clear whether gibbon craniofacial diversity has been kept at a low level through stabilizing selection and/or gene flow across species [14], or whether humans and chimpanzees have diverged anatomically more than expected based on their divergence time, hence pointing to species-specific selective pressures. Either way, the low level of craniofacial diversity observed in hylobatids makes them a useful ‘control’ clade against which variation within hominids can be compared to infer whether humans have evolved in an unusually fast way. Indeed, based on their taxonomic diversity, timing of diversification, frequent hybridization and reduced sexual dimorphism, hylobatids have been claimed to be appropriate evolutionary models for fossil hominins [15] (species that are more closely related to humans than to chimpanzees). Beyond chimpanzees and humans, these comparisons can be expanded to all the great apes, as they all show typical species-specific cranial morphologies that may have evolved in response to particular selective pressures [16]. Indeed, genetic analyses indicate that different species and subspecies of great apes have evolved population-specific genetic adaptations to their habitats [17].

Our understanding of the evolutionary processes that have driven hominoid craniofacial diversification is limited, as is the understanding of the selective factors shaping this diversity. Previous studies have explored the effect of integration on hominid craniofacial evolution [18–21], and others have measured the correlation between anatomical and genetic diversity [22]. Other studies have looked at the differences in developmental trajectories between humans and the great apes [23]. Also, some studies have assessed craniofacial diversity across primates [24], but very few have quantified the tempo and mode of craniofacial evolution in hominins [25–27], hominoids [16,28] or other primates [29]. Those studies have focused on a small number of anatomically homologous landmarks, leaving vast craniofacial regions undescribed. Conversely, detailed analyses of craniofacial evolution using high-density geometric morphometric approaches do not include humans, and their broader evolutionary scale makes it difficult to assess finer scale evolutionary trends within particular groups of primates [30]. To properly describe the tempo and mode of hominoid craniofacial diversity, as well as to understand the evolutionary pressures of the face versus the neurocranium, we used high-density three-dimensional geometric morphometric approaches to measure evolutionary rates for overall craniofacial morphology and for specific craniofacial regions along the evolutionary branches leading to most extant species of the hominoid phylogeny. In doing so, we aimed to detect whether there are fundamental differences in the evolutionary dynamics observed in the great and lesser apes, and whether humans have evolved their craniofacial morphology in a way that departs from that observed in the other apes.

A clear understanding of the patterns of long-term evolution across hominoids is obscured by the different levels of sexual dimorphism across the apes [31]. While some ape species, such as gorillas and orangutans, are highly sexually dimorphic, other species, such as gibbons, chimpanzees and humans, are minimally dimorphic [32]. These differing levels of sexual dimorphism are associated with social differences across species and mediated by hormonal factors that influence cranial development (reviewed in [33]). To understand the relationship between overall selective pressures, long-term evolution and sexual selection, we quantified sex-specific evolutionary trends in craniofacial evolution across the apes. A similar strategy has been used to compare brain evolution between male and female primates and to infer the relationship between brain evolution and behavioural and social evolution [34].

## 2. Results

### (a) Morphological differences and disparity

**Figure 1.**
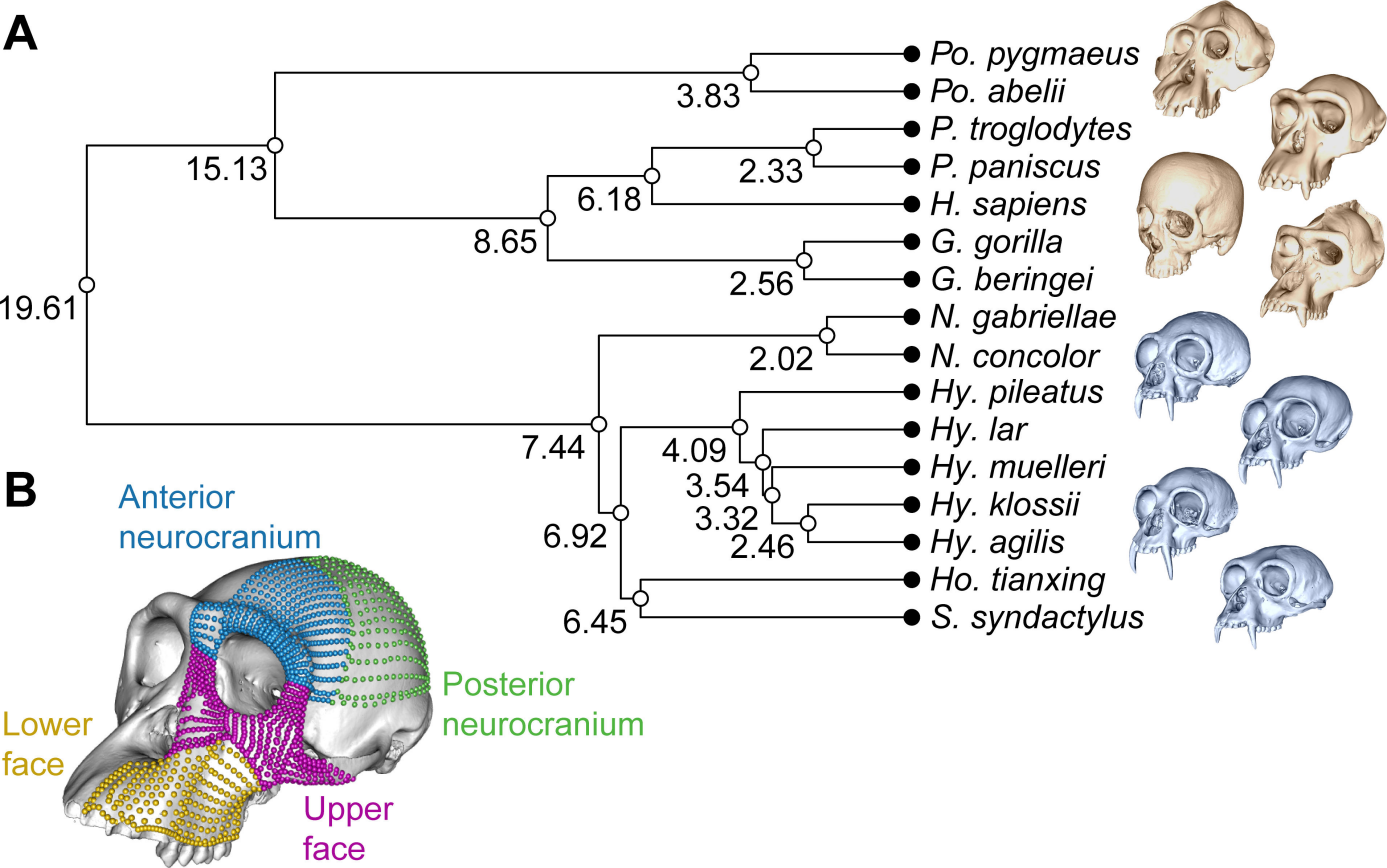
Phylogeny and configuration of landmarks and semilandmarks. (A) Time-calibrated hominoid phylogeny used for analyses indicating node ages (in Ma). One cranium per genus is represented (not to scale), with hominids shaded in orange and hylobatids shaded in blue (from top to bottom: *Pongo, Pan, Homo, Gorilla, Nomascus, Hylobates, Hoolock, Symphalangus*). (B) Chimpanzee model showing the studied configuration of landmarks and semilandmarks, with the different cranial regions represented in different colours: posterior neurocranium, green; anterior neurocranium, blue; upper face, purple; lower face, yellow.

High-density three-dimensional geometric morphometric configurations of landmarks, curves and surface semilandmarks were used to describe craniofacial variation across nine species of hylobatids and seven species of hominids (figure 1A; electronic supplementary material, table S1). This configuration of landmarks and semilandmarks was studied as a whole, and after separation into four different craniofacial regions: posterior neurocranium, anterior neurocranium, upper face and lower face (figure 1B). Evolutionary trends were quantified separately for males and females and then compared.

**Figure 2.**
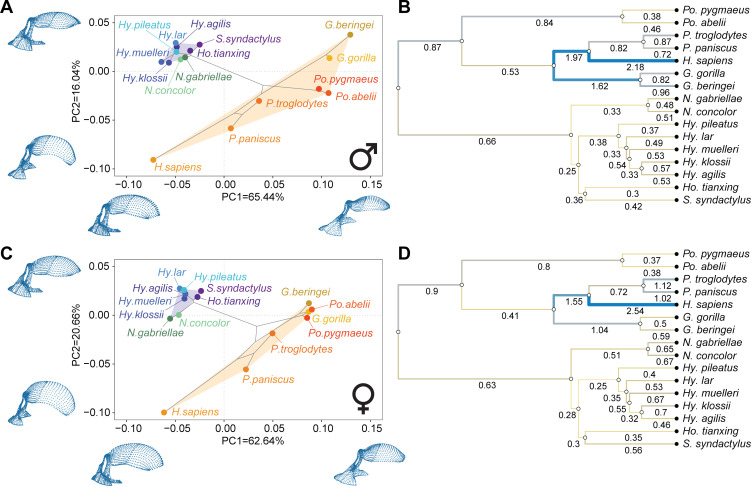
Principal component analyses (PCA) of overall craniofacial variation and corresponding evolutionary rates overlaid on the hominoid phylogeny. (A) PCA plot of male shape variation. (B) Phylogenetic tree with overlaid branch-specific rate values for males. (C) PCA plot of female shape variation. (D) Phylogenetic tree with overlaid rate values for females. In (A) and (C), landmark plots next to and underneath PCA plots show the patterns of variation corresponding to the extreme values of each principal component (PC) within the observed range of variation. In (B) and (D), numbers underneath branches of the phylogenies indicate evolutionary rates for each branch measured as excess change with respect to a neutral expectation, with yellow representing small change (<1), grey representing expected change (≈1), and blue representing large change (>1). Branch thickness is proportional to the evolutionary rate.

Principal component analyses (PCA) of overall craniofacial variation show large craniofacial diversity within hominids, with humans plotting closer to the gibbons than to the great apes owing to their small faces and more globular neurocrania. This general pattern of variation is very similar in both males and females, although male hominids are relatively more spread out across the morphospace with respect to hylobatids than females (figure 2A,C). Posterior neurocranial variation shows a more intermediate position of humans between gibbons and gorillas, whose well-developed cranial crests drive their separation from the other groups, particularly in males (electronic supplementary material, figure S1A,B). Anterior neurocranial variation in males is driven by the separation between humans and gorillas (electronic supplementary material, figure S1C), whereas in females it is more clearly driven by the differences between great apes and gibbons (electronic supplementary material, figure S1D). Upper and lower facial variation is driven by the differences between great apes and gibbons, both in males and females, although humans show up as hominid outliers in all the facial analyses (electronic supplementary material, figure S1E,F,G,H).

Quantification of levels of morphological disparity between hominids and hylobatids shows significant differences for all craniofacial regions in both males and females, although with generally higher disparity ratios (i.e. the ratio between hominid disparity and hylobatid disparity) in males than in females. Indeed, the disparity ratio for overall craniofacial morphology is 9.88 in males (that is, hominids are 9.88 times more variable than hylobatids) and 6.76 in females. The disparity ratio in the posterior neurocranium is 8.47 for males and 2.76 for females; for the anterior neurocranium, the ratio is 5.56 for males and 4.63 for females; for the upper face, the ratio is 2.96 in males and 2.90 in females; for the lower face, the ratio is 2.58 in males and 3.13 in females (table 1). These values show that the neurocranium of male hominids is much more variable than the neurocranium of male hylobatids, whereas the neurocranium of female hominids, while still significantly more variable than that of female hylobatids, is relatively less variable in comparison with males. However, disparity ratios for facial regions are very similar between males and females, indicating that there is no relative increase in facial variation in male hominids with respect to hylobatids in comparison with females.

**Table 1.** Comparison of morphological disparity (measured as Procrustes variance, and as pairwise Procrustes distances) and evolutionary rates (measured as branch-specific excess change with respect to a neutral expectation, and as Brownian motion evolutionary rates) between hominids and hylobatids for all craniofacial regions, and for males and females. For the four variables, a ratio is provided between the value observed in hominids and hylobatids, and a *p*-value for the differences between both clades, which is based on permutation tests for Procrustes variance and Brownian motion rates, and on Wilcoxon rank sum exact tests for pairwise Procrustes distances and excess change. Additional details are provided in the ‘Material and methods’ section.

		morphological disparity		evolutionary rates	
		Procrustes variance ratio (*p*‐value)	pairwise Procrustes distance ratio (*p*‐value)	excess change ratio (*p*‐value)	Brownian motion rate ratio (*p*‐value)
male	whole	9.88 (*p* < 0.001)	3.00 (*p* < 0.001)	2.31 (*p* < 0.001)	4.12 (*p* < 0.001)
	posterior neurocranium	8.47 (*p* < 0.001)	2.84 (*p* < 0.001)	2.20 (*p* < 0.001)	3.35 (*p* < 0.001)
	anterior neurocranium	5.56 (*p* = 0.002)	2.32 (*p* < 0.001)	1.87 (*p* = 0.001)	2.71 (*p* = 0.002)
	upper face	2.96 (*p* < 0.001)	1.69 (*p* < 0.001)	1.39 (*p* = 0.123)	1.43 (*p* = 0.108)
	lower face	2.58 (*p* < 0.001)	1.61 (*p* < 0.001)	1.44 (*p* = 0.053)	1.43 (*p* = 0.131)
female	whole	6.76 (*p* = 0.002)	2.41 (*p* < 0.001)	1.90 (*p* = 0.006)	3.11 (*p* = 0.002)
	posterior neurocranium	2.76 (*p* = 0.008)	1.64 (*p* < 0.001)	1.57 (*p* = 0.053)	1.93 (*p* = 0.018)
	anterior neurocranium	4.63 (*p* = 0.004)	2.07 (*p* < 0.001)	1.57 (*p* = 0.035)	1.98 (*p* = 0.016)
	upper face	2.90 (*p* = 0.007)	1.69 (*p* < 0.001)	1.41 (*p* = 0.103)	1.28 (*p* = 0.266)
	lower face	3.13 (*p* = 0.001)	1.77 (*p* < 0.001)	1.51 (*p* = 0.059)	1.38 (*p* = 0.216)

### (b) Evolutionary rates

A Brownian motion (BM)-based comparison of tree-wide evolutionary rates across the different cranial regions along the whole hominoid phylogeny (including hominids and hylobatids) shows that the posterior neurocranium is the fastest evolving region in males (*σ*^2^_mult_=1.77 × 10^7^), followed by the lower face (*σ*^2^_mult_=1.36 x 10^7^), anterior neurocranium (*σ*^2^_mult_=1.11 x 10^7^) and upper face (*σ*^2^_mult_=0.64 x 10^7^). These differences are significant (*p* = 0.002), with a ratio between the maximum and the minimum rate of 2.74. The posterior neurocranium is also the fastest evolving region in females (*σ*^2^_mult_=1.22 × 10^7^), followed by the lower face (*σ*^2^_mult_=1.02 × 10^7^), anterior neurocranium (*σ*^2^_mult_=0.81 × 10^7^) and upper face (*σ*^2^_mult_=0.49 × 10^7^), with significant differences across regions (maximum to minimum rate ratio = 2.47; *p* = 0.002; electronic supplementary material, table S2).

**Figure 3.**
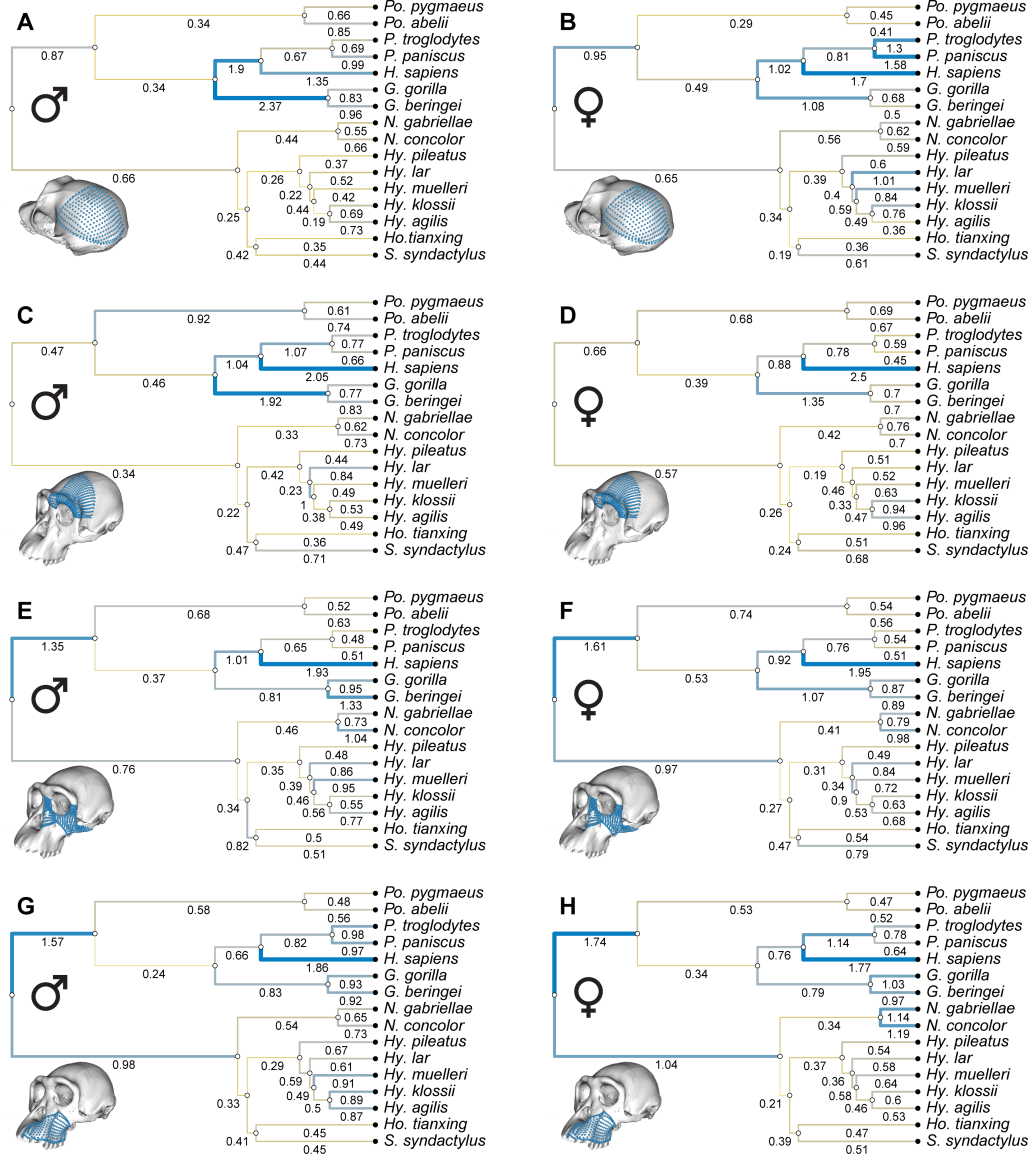
Evolutionary rates observed for each branch of the hominoid phylogeny and each craniofacial region. (A) Posterior neurocranium in males. (B) Posterior neurocranium in females. (C) Anterior neurocranium in males. (D) Anterior neurocranium in females. (E) Upper face in males. (F) Upper face in females. (G) Lower face in males. (H) Lower face in females. Numbers underneath branches indicate evolutionary rates for each branch measured as excess change with respect to a neutral expectation, with yellow representing small change (<1), grey representing expected change (≈1) and blue representing large change (>1). Branch thickness is proportional to the evolutionary rate.

Analysis of branch-specific evolutionary rates (measured as excess change with respect to a neutral expectation) shows that humans have the highest rate across all hominoids for both males and females in all craniofacial regions but the posterior neurocranium in males (figures 2B,D and 3). For all cranial regions, both in males and females, humans tend to accumulate twice as much change as expected if all the species in the hominoid phylogeny had evolved at the same rate. For the neurocranium, the second highest rate tends to be that leading to both species of gorillas, whereas the second highest rate for facial regions tends to be allocated to the basal branch leading to all the great apes. All hylobatid branches show low rates that are below 1, and in most cases below 0.5, thus indicating stabilizing selection for craniofacial morphology within the hylobatid clade (figure 3).

**Figure 4.**
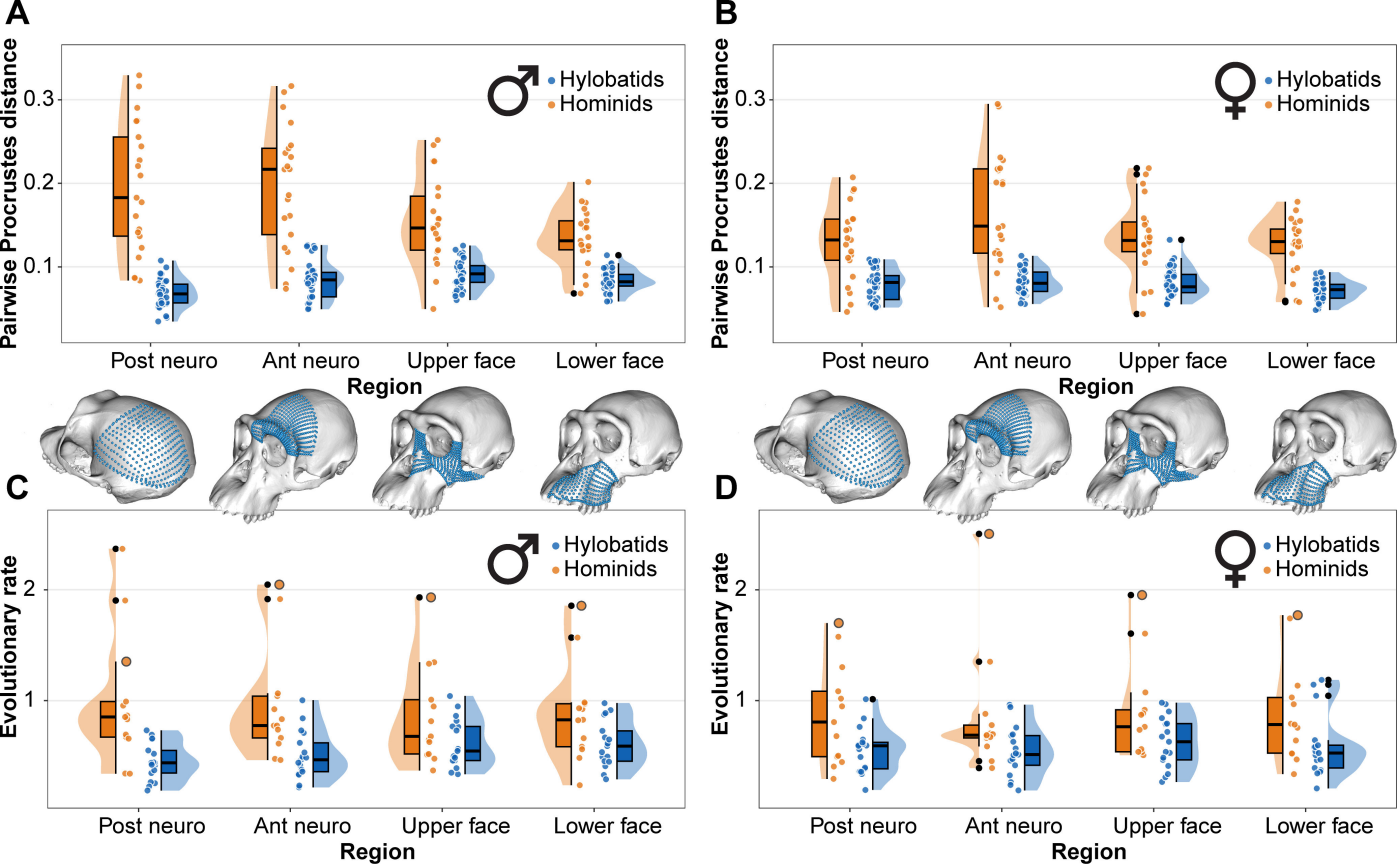
Comparison of morphological disparity and evolutionary rates between hylobatids and hominids for the four studied craniofacial regions. (A) Morphological disparity measured as pairwise Procrustes distances within each clade in males. (B) Morphological disparity measured as pairwise Procrustes distances within each clade in females. (C) Evolutionary rates measured as excess change with respect to a neutral expectation for each branch in males. (D) Evolutionary rates measured as excess change with respect to a neutral expectation for each branch in females. In (C) and (D), the data points corresponding to the human rate for each craniofacial region and sex are highlighted with a black circle.

Branch-specific evolutionary rates are significantly higher in hominids than in hylobatids in neurocranial regions, both in males and females, but these rates do not significantly differ in facial regions (figure 4; table 1). Humans still show a substantially higher evolutionary rate for facial variation than the other hominids, but they emerge as outliers against the other hominid rates, which are generally similar to the hylobatid facial rates and also indicative of evolutionary stasis for most branches (figure 4C,D). Despite the similar rates for facial evolution between hominids and hylobatids, facial disparity is still higher in hominids, but this higher disparity is achieved through similarly slow evolution along the longer branches of the hominid phylogeny (figure 4). By contrast, higher neurocranial disparity in hominids is achieved through additional selection, increasing the diversity of the neurocranium with respect to the face, particularly in some of the hominid branches. These differences are consistent between males and females, but amplified in the male neurocranium.

**Figure 5.**
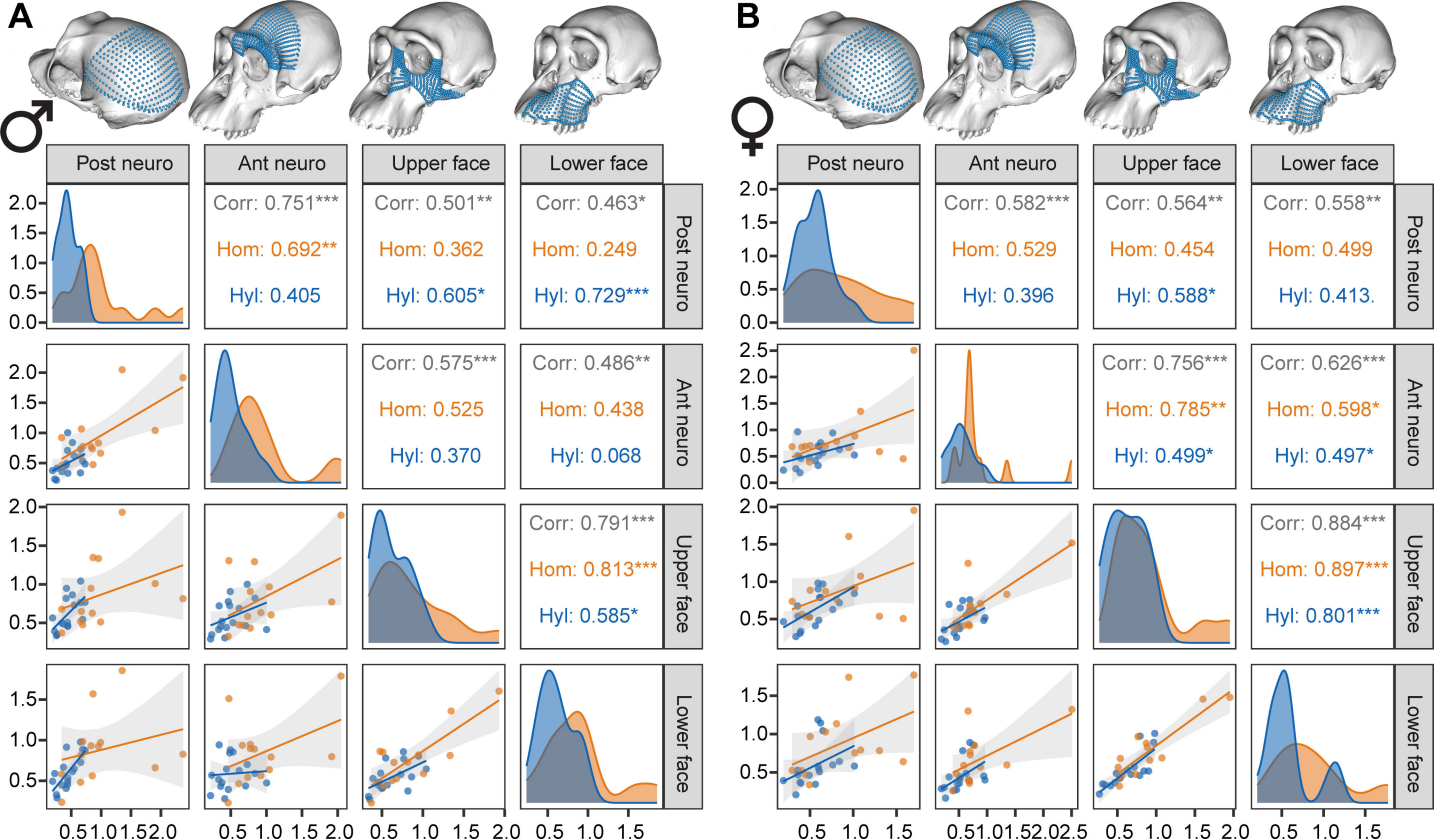
Correlations between evolutionary rates for the four craniofacial regions for hylobatids and hominids. (A) Males and (B) females. Corr, correlation obtained for the combined hylobatid–hominid sample; Hom, correlation found in hominids; Hyl, correlation found in hylobatids. Asterisks indicate significant correlations at *p* < 0.05 (*), *p* < 0.01 (**) or *p* < 0.001 (***).

Patterns of evolutionary integration can be explored by measuring the correlations between rates for the different craniofacial regions. Within hominids, males show a clearer regionalization between neurocranium and face (figure 5A), and females show a generally stronger facial integration and a face that is also strongly integrated with the anterior neurocranium (figure 5B). Hylobatids show a less clear pattern of evolutionary integration, without a clear regionalization of neurocranium and face in males or females (figure 5A,B).

## 3. Discussion

### (a) Accelerated evolution in humans

Our results show that humans have the fastest evolutionary rate across all hominoids in most craniofacial regions and in both males and females. These evolutionary rates are indicative of selection along the human branch, such that humans have accumulated approximately twice as much change as expected under a constant-rate neutral model of evolution across the whole hominoid phylogeny. These results are consistent with previous reports of directional selection across the human branch, with basicranial flexion, cranial vault expansion and facial retraction characterizing the divergence of *Homo* [28]. Our high-density region-by-region approach confirms these results, although it also shows overall differences in the evolution of particular craniofacial regions as described below.

The only craniofacial region where humans do not show the highest evolutionary rate across all hominoids is the posterior neurocranium in males. For the posterior neurocranium, the massive anatomical reorganization associated with the development of cranial crests in male gorillas is associated with the fastest evolutionary rate and ‘dwarfs’ the evolutionary change associated with parietal expansion in humans. This is unexpected because parietal reorganization is considered one of the hallmarks of modern human brain evolution [2,35,36] and a change that is intimately linked to the emergence of our species’ cognitive capacities [3]. As such, parietal expansion is expected to be exposed to strong selection in humans compared with other craniofacial regions. However, our results indicate that the development of cranial crests in male gorillas, a trait that is probably linked to their social organization [37], entails a stronger selective pressure on neurocranial variation than the neurocognitive traits associated with parietal expansion in modern humans.

The evolutionary rates measured for each branch of the hominoid phylogeny can be considered ‘flattened’ values that summarize all the rates corresponding to the fossil record of a given branch. For example, previous studies indicate that evolutionary rates for the shape of teeth and brains are statistically indistinguishable across fossil hominin species, at least as described through two-dimensional geometric morphometric configurations of landmarks and semilandmarks, and through simple linear metrics [38]. Dense configurations of endocranial three-dimensional surface semilandmarks, however, have revealed high evolutionary rates in modern humans and Neanderthals [39,40], but the small number of hominin species included in these studies of endocranial shape does not allow for a finer-grained dissection of the evolutionary rates and modes associated with each period of hominin evolution. Studies of craniofacial variation including a larger number of fossil hominins, together with extant hominoids, indicate potentially different selective regimens [16], but these studies do not include estimates of branch-specific evolutionary rates, nor do they identify individual branches where selection might have happened.

Our finding of strong selection along the human branch may appear contradictory with published studies indicating a predominant role of neutrality in shaping craniofacial diversity in modern humans [41–43]. However, these studies look at different evolutionary scales. While our results show accelerated evolution and directional selection driving craniofacial differences between the species-specific shape of humans and the apes, studies emphasizing neutrality focus on the diversity of present-day modern human populations. Microevolutionary studies of craniofacial diversity among modern human populations and macroevolutionary studies of hominid divergence might be potentially bridged through the incorporation of fossil hominin species, which are not included in our analyses. Previously published analyses of craniofacial variation in fossil hominins indicate that genetic drift can explain most of the changes observed throughout the hominin phylogeny [27], including the differentiation between Neanderthals and modern humans [26], but that selection seems to have a role in the origin and diversification of the genus *Homo* [27,44]. These analyses are based on simple configurations of anatomically homologous landmarks, which brings up the question of whether the use of dense configurations of surface semilandmarks may reveal evolutionary patterns that are unaccounted for when using simpler configurations of homologous landmarks, or whether published studies of fossil hominins and our study of craniofacial evolution across hominoids are measuring variation at different evolutionary scales and results should not be expected to match.

Indeed, there is substantial debate on the opportunities and limitations of using high-density configurations of surface semilandmarks in evolutionary studies [45,46]. When it comes to studying craniofacial variation in hominoids, there are vast anatomical regions, particularly in the neurocranium, that remain undescribed when using only anatomically homologous landmarks; therefore, our study benefits from the use of dense configurations of surface semilandmarks. While the risk of modelling variation that does not have a biological origin does exist [47], this risk is minimal in our study, as only complete and well-preserved specimens were included in our samples. In addition, our methodological approach based on the use of PCA and comparisons of observed versus expected shape distances between estimated ancestors and descendants also overcomes some of the issues associated with the use of high-density geometric morphometrics to study morphological integration ([46]; see Materials and methods). Overall, the use of dense configurations of surface semilandmarks in our study allows for a more accurate description of craniofacial variation across hominoids and provides a clear visualization of the long-term evolutionary trends associated with each craniofacial region. Likewise, we anticipate that the use of dense configurations of three-dimensional surface semilandmarks will be able to reveal selective trends within the hominin fossil record that currently remain undocumented.

### (b) Overall stasis in ape craniofacial evolution, especially in gibbons

Our analyses indicate that most branches of the hominoid phylogeny underwent evolutionary stasis for craniofacial morphology [28,48]. Hylobatids tend to show particularly low evolutionary rates, indicating that the evolution of the hylobatids has been highly conservative [49] and that the whole clade has experienced stabilizing selection with respect to craniofacial shape. However, craniofacial size may have evolved in a faster way in association with the evolution of overall dwarfism in body size [28,50]. The identification of overall evolutionary stasis for gibbon craniofacial shape is not surprising given the craniofacial similarity across all hylobatid species [8] and their rapid adaptive radiation [14]. However, it does point to factors that prevented evolutionary diversification once this initial radiation was complete at approximately 6 Ma [12]. These factors can include stabilizing selection owing to their relatively narrow geographical distribution (in comparison with the great apes) and similar ecological niches in the forest canopy and terminal branches [51], with a preponderance of frugivory [49], and similar social organization across species, with an overall suit of traits comprising small group size, territorial behaviour and monogamy with long-term pairing [49]. Low craniofacial diversity in the hylobatids can also result from gene flow between different species [13], which has been observed in present-day hylobatids, even across genera [52]. While intergeneric hybrids are likely to be sterile because of major differences in chromosome numbers across genera [53], interspecific hybrids within hylobatid genera occur in the wild and can result in fertile hybrids, thus precluding morphological diversification [54].

Our results point to an important distinction between genetic and phenotypic (anatomical) evolution across the apes. While species-specific genetic adaptations have been identified in different ape species [12,55], our results indicate an overall pattern of morphological stasis in craniofacial evolution, with the exception of the human branch. This pattern is particularly interesting in the gibbon clade, where very low craniofacial diversity is maintained in the face of major chromosomal rearrangements across hylobatid genera [14]. While these chromosomal rearrangements may have a major role as a reproductive barrier across hylobatid genera [52,53], they have evolved in spite of the low level of genetic diversity across genera [13] and they do not seem to result in major visible phenotypic changes.

In the case of humans, it would be important to explicitly test whether their high rate of craniofacial evolution tracks a similarly high evolutionary rate for genes involved in craniofacial development [12,55]. However, the contribution of individual genes to overall craniofacial diversity is probably moderate, as overall craniofacial diversity is the result of the complex interaction of a large number of loci. This potential polygenicity is commonly highlighted in genome-wide association studies of complex phenotypes with poorly understood genetic architecture [56]. Further complicating this picture, non-coding regions are also known contributors to craniofacial evolution and diversification [57]. Some of these non-coding regions are fast-evolving developmental enhancers found near genes associated with human facial variation, and they may drive different expression patterns in embryonic development in chimpanzees and humans [58,59]. Lineage-specific accelerated regions are also found in other apes and primates [60,61], some of them with potential phenotypic effects on limb development in gibbons [61], but their potential effect on craniofacial evolution remains to be tested. In any case, our results are consistent with other studies of craniofacial diversity across the great apes, and with the notion that overall integration and strong covariation across craniofacial regions constrain evolutionary diversification to maintain functionality [18,62].

### (c) Evolutionary regionalization of neurocranium and face

Despite this overall integration, our results show a certain degree of evolutionary regionalization in the great ape cranium, which is not observed in the hylobatid cranium. For the great apes, we find a clearer evolutionary regionalization of the neurocranium versus the face. This regionalization is based on the relationship between branch-specific evolutionary rates and does not necessarily reflect developmental integration [63,64]. Rather, this regionalization reflects different patterns of evolution across different anatomical regions, which, in principle, can emerge from differentiated developmental pathways or from different evolutionary dynamics associated with their different functions [65]. Given the overall integration patterns between neurocranium and face observed within hominoid species [20,66] and in other clades [62,67,68], it seems likely that the evolutionary regionalization between neurocranium and face observed throughout great ape evolution occurs because of different selective pressures [69]. This hypothesis is supported by the observation that high morphological disparity in the great ape neurocranium with respect to the hylobatids is associated with significantly higher evolutionary rates, whereas high morphological disparity in the great ape face with respect to the gibbons seems to result simply from genetic drift along the longer branches of the great ape phylogeny. Therefore, our results indicate that the great ape neurocranium is subject to selective pressures that do not influence the great ape face [69]. While it is tempting to link these selective pressures to neurocognitive traits driving great ape evolution, there can also be social factors underlying the fast evolution of the hominid neurocranium, as evidenced by the high evolutionary rate observed in the posterior neurocranium of male gorillas, which is associated with the development of strong cranial crests [37].

While great ape facial evolution can be explained based on stochastic processes, human facial evolution does show a directional trend which matches that observed in the neurocranium (although facial variation across modern human populations seems to result from genetic drift [42]). While there is some evidence that neurocranial and facial directional trends are developmentally associated in humans [5], it is also possible that neurocranial evolution responds to specific neurocognitive selective pressures [2,3], whereas facial evolution responds to specific social and/or environmental selective pressures, including climatic factors [70,71] and social factors related to self-domestication [72]. Because of the lack of fine-grained resolution of our analyses within the hominin clade, it is not possible to assess whether selective pressures on the neurocranium and the face operated exactly within the same hominin lineages, which would point to a certain degree of developmental integration, or whether those selective pressures are temporally decoupled, as it has been shown for dental evolution and brain evolution [38].

### (d) Sex-specific evolutionary trends

Our separate analyses of female- and male-specific evolutionary trends allow us to infer some of the mechanisms influencing the evolution of sex-specific morphologies. In general, we identified higher levels of disparity in males as compared with females, particularly in the great apes. This higher level of morphological disparity within male great apes is consistent with a higher correlation between genetic and anatomical diversity described in the hominoid male neurocranium as compared with females [22]. These results indicate that males are the ones who realize the entire potential for craniofacial diversity encoded in their genomes, while female craniofacial diversity is more constrained. It has been suggested that high levels of oestrogen in prepuberal females make them halt skeletal growth earlier than males [73], which could explain why skeletal traits that develop late, such as cranial crests, are expressed at higher frequencies and degrees of expression in males [37]. This could suggest that it is an earlier growth cessation associated with an earlier attainment of sexual maturity that constrains female craniofacial variation [33]. This hypothesis is supported by the observation that taxa with a higher degree of sexual dimorphism are those where females reach sexual maturity substantially earlier than males (female *Gorilla gorilla*: 7.7 years old versus male *Gorilla gorilla*: 11 years old, [74]; female *Pongo pygmaeus*: 7 years old versus male *Pongo pygmaeus*: 9.6, [75]), whereas males and females reach sexual maturity at similar ages in taxa with low sexual dimorphism (female *Pan troglodytes*: 8 years old versus male *Pan troglodytes*: 9.2 years old; female *Homo sapiens*: 13 years old versus male *Homo sapiens*: 14 years old, [75]). In addition, gorilla and orangutan males maintain growth and development of sexual differences after maturity, further maximizing differences between males and females [37,76]. While our study shows similar patterns of variation and evolutionary dynamics between female and male hominoids, it also reveals sex-specific evolutionary trends that are driven by developmental factors ultimately linked to social organization.

Overall, our results provide the basis for future studies of craniofacial evolutionary rates in fossil hominins. Through these studies, it will be possible to partition the high evolutionary rates found in extant humans into finer-grained evolutionary rates associated with specific branches of the hominin phylogeny. In addition, our approach can be expanded to assess the tempo and mode of evolution of the postcranial skeleton and their association with ecological and behavioural factors [77].

## 4. Material and methods

### (a) Materials

Seven species of hominids (*Homo sapiens, Pan troglodytes, Pan paniscus, Gorilla gorilla, Gorilla beringei, Pongo pygmaeus* and *Pongo abelii*) and nine species of hylobatids (*Symphalangus syndactylus, Hoolock tianxing, Hylobates agilis, Hylobates klossii, Hylobates muelleri, Hylobates lar, Hylobates pileatus, Nomascus concolor* and *Nomascus gabriellae*) were studied. Hominid datasets came from [78] and included between *n* = 7 (*Pongo abelii*) and *n* = 94 (*Pan troglodytes*) individuals per species (electronic supplementary material, table S1). Gibbon samples were obtained for the purposes of this study, and they included four individuals per species (two males and two females). Three-dimensional surface models were obtained from computed tomography scans, which were sourced from NESPOS (https://archiv.neanderthal.de/data/) and MorphoSource (https://www.morphosource.org/), or obtained at the Natural History Museum (London, UK) and Royal Museum for Central Africa (Tervuren, Belgium). Specimens from MorphoSource and NESPOS came originally from the Smithsonian National Museum of Natural History (Washington, USA), American Museum of Natural History (New York, USA) and Department of Anatomy of the University of Leipzig (Leipzig, Germany) (electronic supplementary material, table S1).

### (b) Phylogeny

A time-calibrated hominoid phylogeny was obtained from 10kTrees [79] to be used for phylogenetic comparative analyses (figure 1A). Relationships between hylobatid genera were manually amended to reflect the most updated views on their phylogenetic relationships, using the topology and divergence times among hylobatid genera proposed by [12]. However, it must be noted that the resolution of the gibbon phylogeny remains problematic owing to their pronounced adaptive radiation within a very short evolutionary time period [12,14].

### (c) Geometric morphometrics

Craniofacial variation was described through a unilateral set of anatomically homologous landmarks, curve semilandmarks and surface semilandmarks (figure 1B). A total of 1475 points were placed on each specimen following the semi-automatic approach described by Bardua and colleagues [80], which includes the manual placement of a landmark template and manual adjustment of curve semilandmarks in Stratovan Checkpoint (https://www.stratovan.com/), as well as the automatic placement of surface semilandmarks using the R package *Morpho* [81]. The chosen configuration of landmarks and semilandmarks describes variation in facial regions and in the upper cranial vault, and it can be separated into four anatomical regions: posterior neurocranium, anterior neurocranium, upper face and lower face (figure 1B). The posterior neurocranium corresponds with the parietal bone and the anterior neurocranium with the frontal bone, including the supraorbital region, but the boundaries of these regions had to be defined based on geometric criteria because sutures were usually not visible in three-dimensional models. Owing to the difficulty identifying individual bones, the face was separated into two regions: the upper face region, including the upper part of the maxillary bone, nasal and zygomatic bones, and the lower face region, including the lower part of the maxillary bone and the premaxilla (figure 1B). This configuration of landmarks and semilandmarks does not include basicranial variation because it was originally designed to describe facial variation and the variation of those neurocranial regions that influence facial variation [78]. Once projected onto individual specimens, curve and surface semilandmarks were slid along their respective tangents to maximize geometric correspondence by minimizing the bending energy for each sample [81]. Generalized Procrustes analysis was performed on complete configurations of landmarks and semilandmarks and on each individual craniofacial region using the R package *geomorph* [82]. Species-specific consensus shapes were calculated for each hominoid species, separating males and females. For those species that have several subspecies, a grand mean was calculated as the species-specific consensus shape.

### (d) Statistical analyses

PCAs of species-specific consensus shapes (Procrustes coordinates) were carried out to assess patterns of variation for each cranial region. Morphological disparity (Procrustes variance) was compared between hominids and hylobatids for each craniofacial region using the R package *geomorph* [82]. Procrustes variance is calculated as the sum of the diagonal elements of the group covariance matrix divided by the number of observations in the group, and it was compared as a variance ratio indicating how many times the shape variance observed in hominids is greater than the shape variance observed in hylobatids for each craniofacial region. Because Procrustes variance provides information only about mean shape disparity within each group, we also calculated distributions of pairwise Procrustes distances between all species within each clade (hominids and hylobatids), which were compared with evolutionary rates within each clade (see below).

Linear modelling in *geomorph* was used to assess the evolutionary relationship between craniofacial shape and size variation, whose measurement should account for phylogenetic signal (*λ*) [83]. However, because of the small number of taxa involved in the comparisons, phylogenetic signal cannot be accurately quantified in our sample [84]. Therefore, the effect of allometry was calculated in two extreme scenarios, when fixing *λ* to 0 (meaning there is no phylogenetic signal in the dataset) and when fixing *λ* to 1 (meaning that all variation in the dataset is explained by phylogeny). Results were very similar across cranial regions and sexes, showing non-significant allometry when assuming *λ* = 1, and significant allometric effects when assuming *λ* = 0, with size variation accounting for between 10% and 70% of shape variation (electronic supplementary material, tables S3 and S4). Therefore, disparity and evolutionary rate analyses were recalculated in allometry-corrected residuals obtained when forcing *λ* = 0, which is the scenario that maximizes the effect of allometry (electronic supplementary material, table S5). These results mostly reproduce the results obtained from allometry-uncorrected shape coordinates in showing significantly higher disparity in hominids than in hylobatids in all craniofacial regions, and higher evolutionary rates in hominids than in hylobatids in the neurocranium, but not in the face (electronic supplementary material, table S5). Results based on allometry-corrected residuals also indicate that humans show the highest rate in almost all craniofacial regions (electronic supplementary material, table S5). Considering that the true value of *λ* falls somewhere between 0 and 1, our results on the differences in disparity and evolutionary rates between hominids and hylobatids are likely to hold regardless of the true value of *λ* and the real effect of allometry on shape variation.

### (e) Evolutionary rates

Branch-specific evolutionary rates were measured as excess change per branch with respect to a constant-rate neutral expectation [38]. To do so, we first used all principal components (PCs) of shape variation to calculate ancestral PC scores for all the nodes of the hominoid phylogeny under a multiple variance Brownian motion (mvBM) approach [85]. An mvBM approach was chosen versus other methods of ancestral state reconstruction because it does not make any assumptions about the tempo and mode of evolution across a given phylogeny and because, unlike other available variable rate approaches, it works well with small phylogenies [85]. The mvBM approach is implemented in the R package *evomap* [86], and it uses local and global information across the phylogeny to estimate ancestral states, converging in a standard BM estimate when evolutionary rates do not differ.

Ancestral PC scores were transformed back into ancestral shapes (as described by landmark and semilandmark coordinates [87]), and Procrustes distances (shape distances measured as the square root of the sum of the squared distances between homologous landmarks and semilandmarks) were calculated between each ancestor and its descendant species [38]. These observed shape distances between ancestors and descendants were compared with expected distances between ancestors and descendants, which were calculated through 100 simulations of craniofacial evolution along the hominoid phylogeny [38,87]. These simulations were carried out in the PC space using functions from the R packages *phytools* [88], *ape* [89] and *geiger* [90], and after calculating a phylogeny-wide per-generation variance parameter from the dataset. This was attained by re-scaling the phylogeny to generations using species-specific generation times [74]. Simulations were run along the hominoid phylogeny for the number of generations inferred for each branch using the per-generation variance parameter calculated for each PC. Ancestral PC scores obtained in these simulations were transformed into ancestral landmark coordinates, and Procrustes distances were calculated between simulated ancestral and descendant shapes across the entire phylogeny. The simulation-based Procrustes distances obtained for a given branch, which depend on the length of that branch, were averaged over 100 simulations and used as the expected Procrustes distance corresponding to that branch. Evolutionary rates were calculated as the ratio between observed and expected (simulated) Procrustes distance for each branch.

The evolutionary rates obtained following this workflow can be interpreted as excess change with respect to a neutral expectation that assumes that all the branches of the phylogeny evolve at the same rate. Rates with a value higher than 1 indicate that branches are accumulating more change than expected under a neutral model and therefore experiencing directional selection. Branches with a rate value lower than 1 are accumulating less evolutionary change than expected and therefore experiencing stabilizing selection [38]. Unlike rates corresponding to traits that can increase or decrease their trait value (e.g. size), rates associated with shape variation are unsigned.

In addition, we used a BM-based approach to compare evolutionary rates across craniofacial regions [91] and between hominids and hylobatids [92] as implemented in the R package *geomorph* [82]. While results obtained when comparing hominid and hylobatid evolutionary rates with mvBM and standard BM approaches generally agreed, the standard BM approach was used to further ascertain the statistical significance of differences when mvBM results yielded borderline *p*-values.

## Data Availability

The scripts and datasets used to carry out analyses are available at [93]. Supplementary material is available online [94].
